# Comparative in vivo biodistribution of cells labelled with [^89^Zr]Zr-(oxinate)_4_ or [^89^Zr]Zr-DFO-NCS using PET

**DOI:** 10.1186/s13550-023-01021-1

**Published:** 2023-08-08

**Authors:** Ida Friberger, Joachim N. Nilsson, Li Lu, Jonathan Siikanen, Oscar Ardenfors, Stefan Milton, Erik Samén, Jeroen A. C. M. Goos, Mattias Carlsten, Staffan Holmin, Thuy A. Tran

**Affiliations:** 1 Department of Clinical Neuroscience, Karolinska Institutet, Stockholm, Sweden; 2 Department of Medical Radiation Physics and Nuclear Medicine, Karolinska University Hospital, Stockholm, Sweden; 3 Department of Molecular Medicine and Surgery, Karolinska Institutet, Stockholm, Sweden; 4 Department of Oncology and Pathology, Karolinska Institutet, Stockholm, Sweden; 5 Department of Radiopharmacy, Karolinska University Hospital, Stockholm, Sweden; 6 Center for Hematology and Regenerative Medicine (HERM), Karolinska Institutet, Stockholm, Sweden; 7 Centre for Cell Therapy and Allogeneic Stem Cell Transplantation (CAST), Karolinska Comprehensive Cancer Center, Karolinska University Hospital, Stockholm, Sweden; 8 Department of Neuroradiology, Karolinska University Hospital, Stockholm, Sweden

**Keywords:** Cell tracking, ^89^Zr, Oxine, Deferoxamine, PET, Dosimetry, Macrophages, DSC

## Abstract

**Background:**

In vivo monitoring of cell biodistribution using positron emission tomography (PET) provides a quantitative non-invasive method to further optimize cell therapies and related new developments in the field. Our group has earlier optimized and evaluated the in vitro properties of two radiotracers,[^89^Zr]Zr-(oxinate)_4_ and [^89^Zr]Zr-DFO-NCS, for the radiolabelling of different cell types. Here, we performed a microPET study to assess the in vivo biodistribution of cells in rats using these two radiotracers. Human decidual stromal cells (hDSC) and rat macrophages (rMac) were radiolabelled with [^89^Zr]Zr-(oxinate)_4_ or [^89^Zr]Zr-DFO-NCS. Rats were intravenously injected with radiolabelled cells, and the in vivo biodistribution was monitored with microPET/CT imaging for up to day 7. Organ uptake was evaluated and presented as a percentage of injected activity per gram tissue (%IA/g) and total absorbed organ doses (mSv/MBq).

**Results:**

The biodistribution in vivo showed an immediate uptake in the lungs. Thereafter, [^89^Zr]Zr-(oxinate)_4_ labelled cells migrated to the liver, while the signal from [^89^Zr]Zr-DFO-NCS labelled cells lingered in the lungs. The differences in the in vivo behaviour for the same cell type appeared related to the radiotracer labelling. After 24 h, [^89^Zr]Zr-(oxinate)_4_ labelled cells had over 70% higher liver uptake for both hDSC and rMac compared to [^89^Zr]Zr-DFO-NCS labelled cells, whereas [^89^Zr]Zr-DFO-NCS labelled cells showed over 60% higher uptake in the lungs compared to [^89^Zr]Zr-(oxinate)_4_ labelled cells. This difference in both lung and liver uptake continued until day 7. Dosimetry calculations showed a higher effective dose (mSv/MBq) for [^89^Zr]Zr-DFO-NCS compared to [^89^Zr]Zr-(oxinate)_4_, for both cell types. Although the bone uptake was higher for [^89^Zr]Zr-(oxinate)_4_ labelled cells, the prolonged uptake in the lungs contributed to a significant crossfire to bone marrow resulting in a higher bone dose.

**Conclusion:**

The [^89^Zr]Zr-DFO-NCS labelled cells suggest a prolonged accumulation in the lungs, while [^89^Zr]Zr-(oxinate)_4_ suggests quicker clearance of the lungs followed by accumulation in the liver. Accumulation of radiolabelled cells in the liver corresponds to other cell-tracking methods. Further studies are required to determine the actual location of the [^89^Zr]Zr-DFO-NCS labelled cell.

**Supplementary Information:**

The online version contains supplementary material available at 10.1186/s13550-023-01021-1.

## Introduction

Based on insights from allogeneic stem cell transplantation and the practice of donor lymphocyte infusions, clinical trials show the anti-tumour potential of tumour-infiltrating lymphocytes and natural killer cells. More recently the success of CAR-T cells (Chimeric Antigen Receptors T cell) for haematological indications, the immune therapy space is currently exploring an array of different cellular immunotherapies for the treatment of cancer [1–3]. Similar approaches also hold promise for non-cancer indications such as autoimmune diseases [2]. However, as infused cells in contrast to non-living drugs in most cases have unknown in vivo distribution, the efficiency of cellular therapies cannot always be fully predicted. This is also true for side effects that are usually caused by the off-target accumulation of the infused cells in the healthy organs. One well-known example of this is graft-versus-host disease (GvHD) [4]. Consequently, to ensure effective treatment while minimizing complications from off-target toxicity is essential to develop reliable methods that can dynamically determine the in vivo biodistribution of the infused cells [5]. Long-term cell tracking with long-lived radionuclide-based tracers can provide the necessary information on cell behaviour and migration in vivo with real-time nuclear imaging [6]. A vast number of radiotracers have been investigated for long-term cell tracking in vivo. The clinically used Single-Photon Emission Computerized Tomography (SPECT) radiotracer [^111^In]In-oxine (also denoted as [^111^In]In-(oxinate)_3_) is suboptimal due to the limited spatial resolution of SPECT in vivo and isotope leakage [7]. Current PET radiotracers are in many ways superior to SPECT regarding half-life, resolution and stability and might provide higher cellular retention. The isotope zirconium-89 (^89^Zr) is an attractive isotope within PET imaging and fulfils several parameters required for cell tracking. Two of the most common chelators developed for ^89^Zr are oxine and DFO-NCS. The main difference between these two radiotracers is the cell labelling mechanism. The [^89^Zr]Zr-(oxinate)_4_ passively diffuses over the cell membrane where the complex dissolves. The 89Zr binds to unspecific molecules inside the cell, primarily in the cytosol, cell membrane, nucleus, chromatin and cytoskeleton [8–10]. The [^89^Zr]Zr-DFO-NCS binds to any free amine available on molecules on the cell membrane surface. Today, [^89^Zr]Zr-(oxinate)_4_ (also denoted as [^89^Zr]Zr-oxine) is a well-evaluated radiotracer in preclinical studies and is currently in a first-in-human clinical study [9, 11–15]. However, some limitations regarding radioactive leakage during the first 24 h should be further evaluated [10, 16, 17]. Our group have recently optimized the synthesis and cell labelling of both [^89^Zr]Zr-(oxinate)_4_ and [^89^Zr]Zr-DFO-NCS [17]. Both radiotracers were successfully synthesized with a radiochemical yield (RCY) of > 95% and used to label different cell types with high labelling efficiency. This study aims to directly compare these two radiotracers in terms of in vivo biodistribution and preliminary dosimetry in rats.

## Materials and methods

### Cell preparations

Human decidual stromal cells (hDSC): The hDSC were generously provided by Dr Helen Kaipe, Karolinska Institutet. They were prepared as previously described [18, 19]. In short, hDSC were isolated from the human placenta, through a caesarean section on healthy donors after informed consent, according to legislation by the Swedish Institutional Ethical Review Board (Dnr: 2009/418–31/4, 2010/2061–32, Dnr: 2015/1848–31/2). The recovered placenta was washed, dissected and cultured until passage 3 or 4 and then gradually frozen. The hDSC were analysed and tested positive for cell-specific antigen expression [19]. Rat macrophages (rMac): Bone marrow-derived macrophages from rats (rMac) were collected in accordance with the ethics approval Dnr: 9328-2019 N138/14, as previously described by Weichenfeldt and Porse [20]. Briefly, the femurs were surgically removed, and the bone marrow (BM) was flushed out. The collected cell mixture was then suspended and cultured for 8 days in complete DMEM (including 20% foetal bovine serum (FBS) (Gibco), 1% streptomycin and 20 ng/ml rat macrophage colony-stimulating factor M-CSF (PeproTech)). The macrophages were detached, resuspended in PBS and analysed with FACS analysis (BD Biosciences or Merck Guava H12) with FACSCalibur software (FlowJo v10, BD Biosciences) as previously described [17, 20].

These two radiotracers are considered universal for all cell types. The purpose of using rMac and hDSC was to include two completely different cell types derived from both rats and humans since different cell types can have different migration patterns in vivo*.* To ensure that the radiotracers do not interfere with the cell behaviour, any cell-specific migration pattern should correlate regardless of which radiotracers are used.

### Radiosynthesis and cell labelling with [^89^Zr]Zr-(oxinate)_4_ and [^89^Zr]Zr-DFO-NCS

^89^Zr was purchased from PerkinElmer or produced in-house with a cyclotron (PETtrace 800, GE Healthcare) with an ^89^Y(p,n)^89^Zr reaction as previously described (Additional file 1: Materials and methods) [17]. Synthesis of [^89^Zr]Zr-(oxinate)_4_ and [^89^Zr]Zr-DFO-NCS with a radiochemical yield of over 95% was obtained according to our previous publication (Additional file 1: Materials and methods) [17]. Optimized protocols for radiolabelling with [^89^Zr]Zr-(oxinate)_4_ and [^89^Zr]Zr-DFO-NCS were previously described (Additional file 1: materials and methods) [17].

### In vitro viability and radioactive retention

The previous study presents an in vitro evaluation of the long-term (7 days) effects on cell viability and radioactive retention inflicted by radiolabelling of hDSC and rMac [17]. Previous studies have established plasma stability of compound binding [13, 21]. In this study, we evaluated the short-term effects on the viability and radioactive retention of radiolabelled rMac. Since previous data suggest that rMac are more sensitive, it was, therefore, suitable that further investigation of rMac can be performed in this study. Radiolabelling was performed in triplicates in which 1.5 ± 1.5 × 10^6^ cells were labelled with [^89^Zr]Zr-(oxinate)_4_ (resulting in 1.3 ± 0.12 MBq/10^6^ cells) and 1.1 ± 0.5 × 10^6^ cells were labelled with [^89^Zr]Zr-DFO-NCS (resulting 1.3 ± 0.38 MBq/10^6^), as well as 1.3 ± 0.23 × 10^6^ unlabelled cells were used as controls. Radiolabelled and unlabelled rMac were cultured in complete DMEM; on days 0, 1, 2 and 4, the cells were measured for radioactive retention, counted and determined viability by Trypan blue staining.

### In vivo studies

Animal handling and experimental procedures were conducted according to the guidelines of the Animal Welfare Board at the Karolinska Institute and were approved by the Stockholm Northern Regional Ethical Committee (5.2.18-10793/16, N4/15, 4043-22). Experiments were conducted and reported in compliance with the Animal Research: Reporting in-Vivo Experiments (ARRIVE) guidelines. Animals were kept in groups of 2–3 in cages, never single-caging, in a humidity-controlled, thermo-regulated facility with a 12-h/12-h light/dark cycle and access to food and water ad libitum. Animals were euthanized through an overdose of anaesthesia followed by mechanical dislocation of the spine. For the imaging experiments, the animals were anaesthetized using an isoflurane/oxygen gas mixture (5% for induction, 1.5–2% for maintenance). The anaesthetic concentration was regulated using an E-Z anaesthesia vaporizer and blended with 6:4 air/O2 (Euthanex Corporation, PA). Body temperature and heart rate were monitored and kept stable by heating and regulated anaesthesia during the scan. A total of 22 male Sprague Dawley rats from Javier (399 ± 64 g, 10–12 weeks) were used, divided into 4 groups; (A) control with neutralized [^89^Zr]Zr-(oxalate)_4_ (7.2 MBq, n = 1), to confirm previous reports [11, 12]. Group (B) controls with unbound radiotracer [^89^Zr]Zr-(oxinate)_4_ (n = 4) or [^89^Zr]Zr-DFO-NCS) (n = 4) and received 5.2 ± 0.96 MBq and 5.1 ± 1.1 MBq, respectively. Group (C) was injected with [^89^Zr]Zr-(oxinate)_4_ radiolabelled hDSC (n = 4) or rMac (n = 3) and received 3.3 ± 0.51 MBq (2.5 ± 0.91 × 10^6^ viable cells) and 1.8 ± 0.49 MBq (0.80 ± 0.10 × 10^6^ viable cells), respectively. Finally, group (D) with [^89^Zr]Zr-DFO-NCS radiolabelled hDSC (n = 3) or rMac (n = 3) and received 3.4 ± 1.5 MBq (2.8 ± 1.8 × 10^6^ viable cells) and 2.3 ± 1.1 MBq (1.5 ± 0.92 × 10^6^ viable cells), respectively. All injected samples were neutralized to pH 7.4 using 1 M sodium carbonate and administered by a slow 30-s intravenous (i.v.) injection through the tail vein of each rat under anaesthesia. Control experiments were performed with injections of unbound [^89^Zr]Zr-(oxalate)_4_, [^89^Zr]Zr-(oxinate)_4_ and [^89^Zr]Zr-DFO-NCS without cells. To prevent binding of unbound [^89^Zr]Zr-DFO-NCS to blood components, the NCS side chain was hydrolysed prior to injection by a 3-h incubation in PBS at pH 8.5. The first PET images were taken 1 min after injection (t = day 0) using the MicroPET Focus 120 scanner (CTI-Concorde Microsystems LLC, Knoxville, TN, USA). PET images were taken in pairs, 45 min each, first upper body and followed by the lower body, which was later merged to provide whole-body images. The imaging was then repeated at 1, 3 and 7 days after injection. PET data were acquired in three-dimensional (3-D) mode, and images were reconstructed by standard 2-D filtered back-projection using a ramp filter. PET data were processed using MicroPET Manager and evaluated using the Inveon Research Workplace (IRW) software (Siemens Medical Systems, Malvern, PA, USA). PET images were also evaluated using an in-house viewer software based on MATLAB by a second observer to validate organ regions-of-interest (ROI) placement [22, 23]. The organs of interest were the lungs, liver, spleen, kidneys, bone (femurs ink. knees) and the whole heart (representing the blood). PET images are decay corrected against the time of injection on day 0 and presented with a %IA/mL unit scale. Blood sampling was not obtainable due to ethical restrictions; the blood pool distribution was instead estimated from the heart ROI. The whole-body activity at time points days 1, 3 and 7 was calculated by the total detected activity in the animal from the PET images, except for the day 0 activity which was measured by the total activity injected.

### Dosimetry

Estimates of absorbed doses to the lungs, liver, spleen, kidneys and bone were calculated from the PET data. The organs were either segmented in their entirety or using representative parts (knees were used to represent all bones). Using these data and the ex vivo-measured organ masses, the injected activity per gram of tissue [%IA/g] was estimated. If organ masses could not be measured ex vivo, organ masses were estimated from average specific organ weights in the full rat cohort, scaled with the corresponding rat whole-body weight. Biodistribution data were analysed and calculated by two independent analysts for later comparison. The calculations on absorbed organs dose and whole-body effective dose are described in Additional file 1: Materials and methods. Data are presented as the mean value with a standard deviation (SD). Statistical significance for two-group single measurements was calculated using the Student’s t test in Excel Office (Microsoft Professional Plus 2019). The rm-ANOVA with Bonferroni multiple corrections was used for groups with repeated measurement. A p-value of < 0.05 was considered as being statistically significant.

## Results

### Radiosynthesis and cell labelling

Synthesis of [^89^Zr]Zr-(oxinate)_4_ and [^89^Zr]Zr-DFO-NCS was performed with a radiochemical yield (RCY) of > 95%. [^89^Zr]Zr-(oxinate)_4_ and [^89^Zr]Zr-DFO-NCS stability and shelf-life after neutralization were determined in a previous study [17]. [^89^Zr]Zr-(oxinate) remained stable after neutralization to pH 7.4, while [^89^Zr]Zr-DFO-NCS risk hydrolysation hence has a short shelf-life.

Cell labelling with [^89^Zr]Zr-(oxinate)_4_ yielded a cell labelling efficiency (CLE) of 54 ± 6.6%, 2.5 ± 0.91 MBq/10^6^ (n = 4) for hDSC and 70 ± 13%, 0.8 ± 0.1 MBq/10^6^ (n = 3) for rMac. Cell count and viability measured with Trypan blue staining disclosed a live cell count of 84 ± 5.6% (1.3–5 × 10^6^ cells) for hDSC and 75.0 ± 8.3% (1.8–3.8 × 10^6^ cells) for rMac. The cell labelling with [^89^Zr]Zr-DFO-NCS provided a CLE of 67 ± 9.6%, 2.8 ± 1.8 MBq/10^6^ (n = 3) for hDSC and 55 ± 20%, 1.5 ± 0.92 MBq/10^6^ (n = 3) for rMac with a viability of 85 ± 2.6% (1.2–2.7 × 10^6^ cells) and 83 ± 3.4% (1.4–4.5 × 10^6^ cells) for hDSC and rMac, respectively.

### Cell viability and short-term radioactive retention

**Fig. 1:**
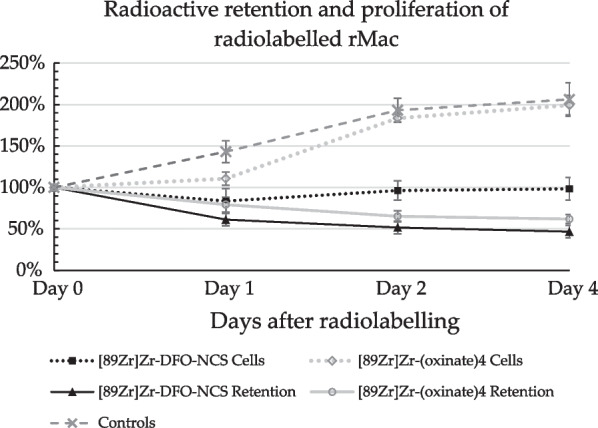
Radioactive retention and proliferation of rMac after radiolabelling with [^89^Zr]Zr-(oxinate)_4_ or [^89^Zr]Zr-DFO-NCS. Rat macrophages (rMac) were radiolabelled in vitro and cultured for 4 days; cells were measured for viability and radioactive retention on days 0, 1, 2 and 4

The previous study showed cellular retention of [^89^Zr]Zr-(oxinate)_4_ dropped during the first 24 h followed by a stabilized radioactive retention until day 7 [17]. With a radioactive cell dose of 1.3 ± 0.12 MBq/10^6^, [^89^Zr] Zr-(oxinate)_4_ labelled rMac showed a drop in radioactive retention with − 16 ± 1.8%, − 31 ± 1.9% and − 34 ± 1.2% on days 1, 2 and 4, respectively (Fig. 1). Macrophages labelled with [^89^Zr]Zr-DFO-NCS showed no significant difference in radioactive retention compared to the [^89^Zr]Zr-(oxinate)_4_ labelled cells (*p* = 0.06). The radioactive retention of ^89^Zr from [^89^Zr]Zr-DFO-NCS labelled cells decreased with − 39 ± 7.6%, − 48 ± 7.6% and − 53 ± 7.5% on days 1, 2 and 4, respectively. Our group has previously reported the radioactive cellular retention from both [^89^Zr]Zr-(oxinate)_4_ and [^89^Zr]Zr-DFO-NCS labelled hDSC and rMac [17]. Unlabelled control rMac showed a steady increase in cell count with + 43 ± 13% on day 1, + 93 ± 14% on day 2 and + 106 ± 20% on day 4. The cell counts for the [^89^Zr]Zr-(oxinate)_4_ labelled cells also increased over time with + 11 ± 7.9%, + 84 ± 4.9% and + 99 ± 12% on days 1, 2 and 4, respectively, with no significant difference compared to unlabelled controls (*p* = 0.07). Labelling with [^89^Zr]Zr-DFO-NCS, however, appeared to have a significant effect on cell proliferation, compared to unlabelled control cells (*p* = 0.03). [^89^Zr]Zr-DFO-NCS labelled cells showed no sign of proliferation; after 24 h, the cell count decreased with − 16 ± 15% and then remained relatively stable at − 3.7 ± 12% on day 2 and − 1.7 ± 14% on day 4.

### PET imaging and biodistribution

**Fig. 2:**
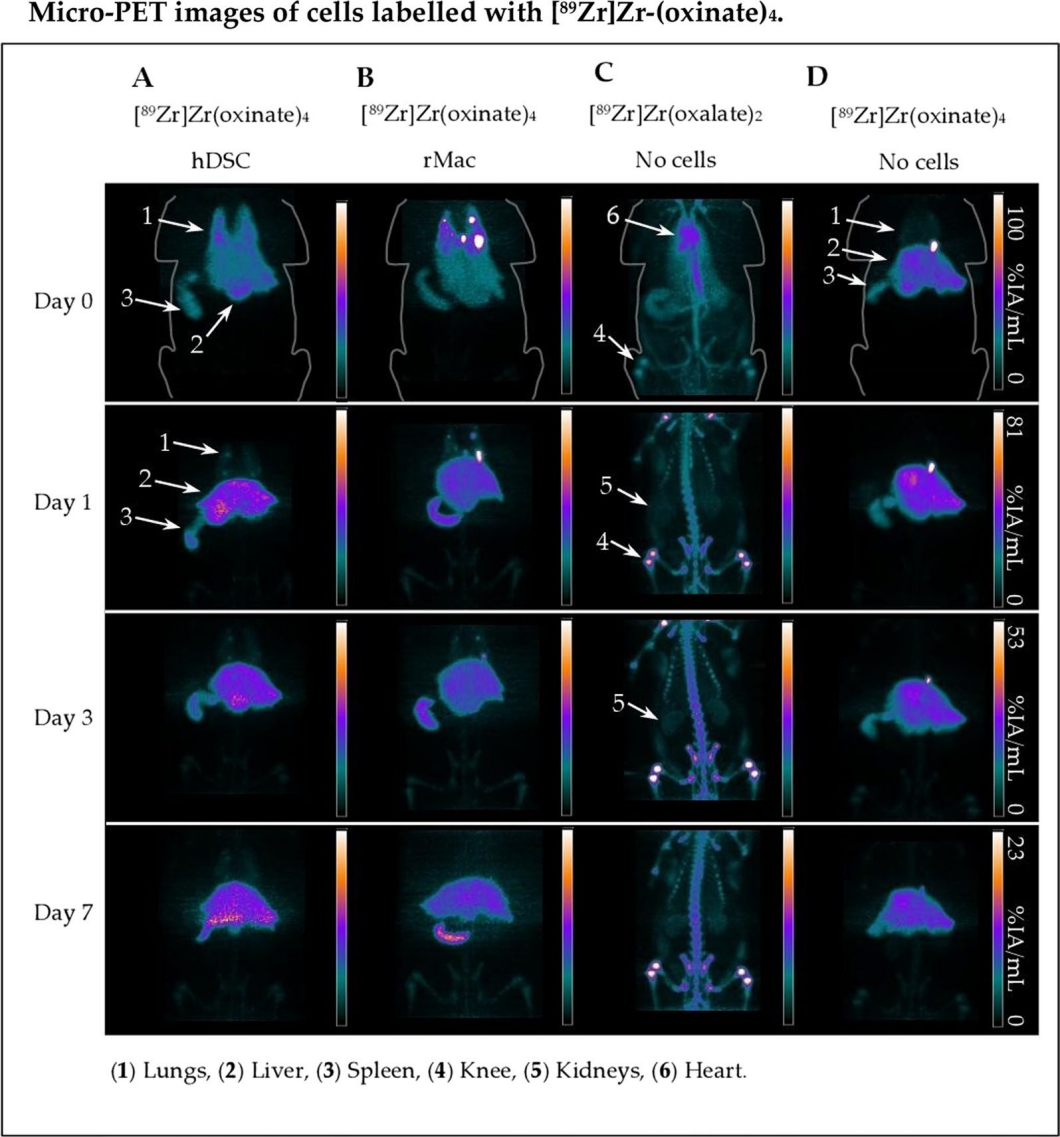
Representative microPET images from iv. transplanted cells labelled with [^89^Zr]Zr-(oxinate)_4_. Healthy male rats were injected with either **a** hDSC or **b** rMac labelled with [^89^Zr]Zr-(oxinate)_4_. Controls were injected with either **c** unbound neutralized [^89^Zr]Zr-(oxalate)_4_ (n = 1) or **d** unbound neutralized [^89^Zr]Zr-(oxinate)_4_. PET images were divided into upper-body and lower-body scans to be later merged into whole-body images. The same colour intensity scale was used for both upper and lower images throughout time. PET images are decay corrected against the time of injection on day 0 and presented with a percentage of injected activity per mL (%IA/mL) scale

**Fig. 3:**
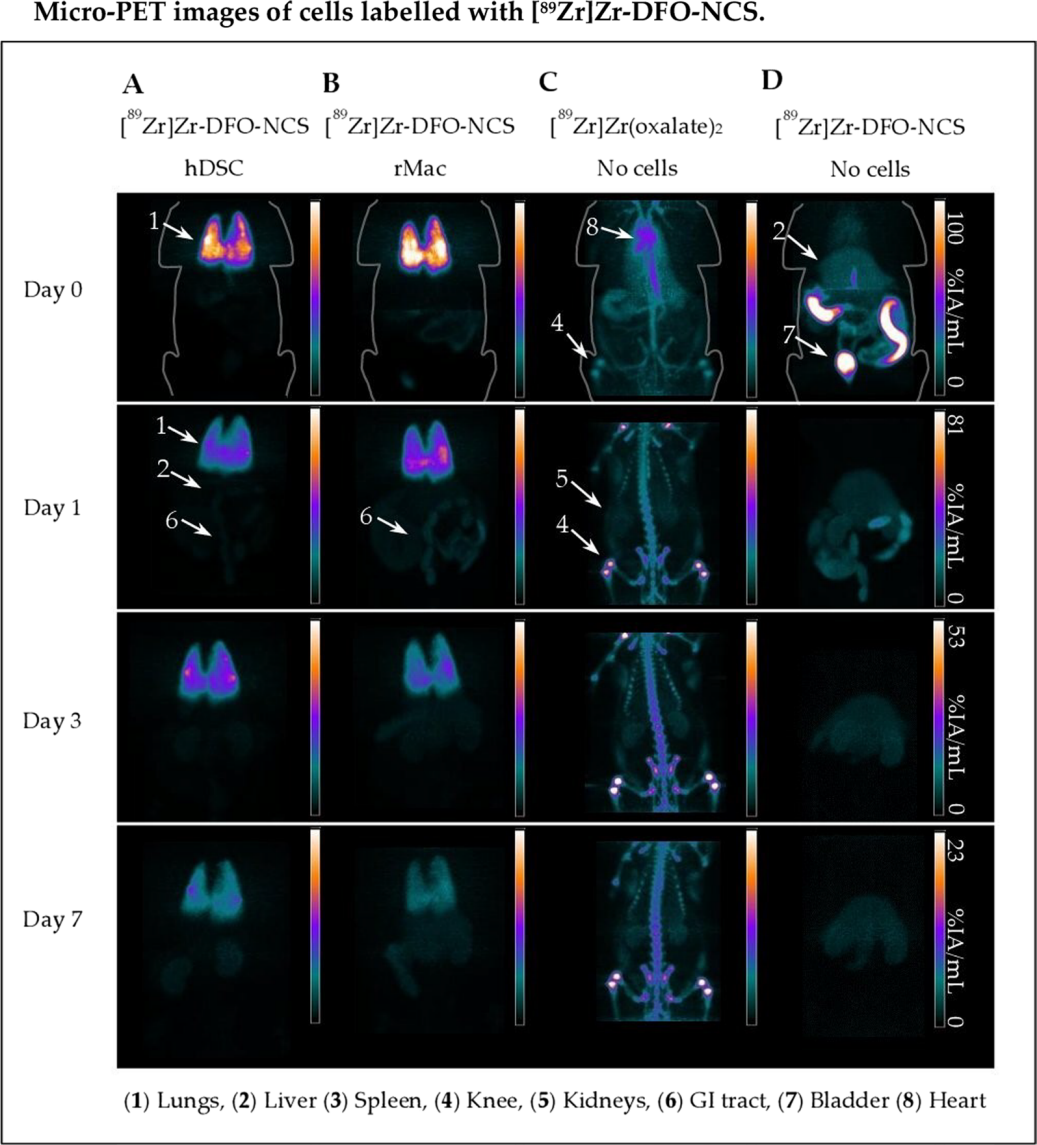
Representative microPET images from iv. transplanted cells labelled with [^89^Zr]Zr-DFO-NCS. Healthy male rats were injected with either **a** hDSC or **b** rMac labelled with [^89^Zr]Zr-DFO-NCS. Controls were injected with either unbound neutralized **c** [^89^Zr]Zr-(oxalate)_4_ (n = 1) or **d** hydrolysed [^89^Zr]Zr-DFO-NCS. PET images were divided into upper-body and lower-body scans to be later merged into whole-body images. The same colour intensity scale was used for both upper and lower images throughout time. PET images are decay corrected against the time of injection on day 0 and presented with a percentage of injected activity per mL (%IA/mL) scale

**Fig. 4:**
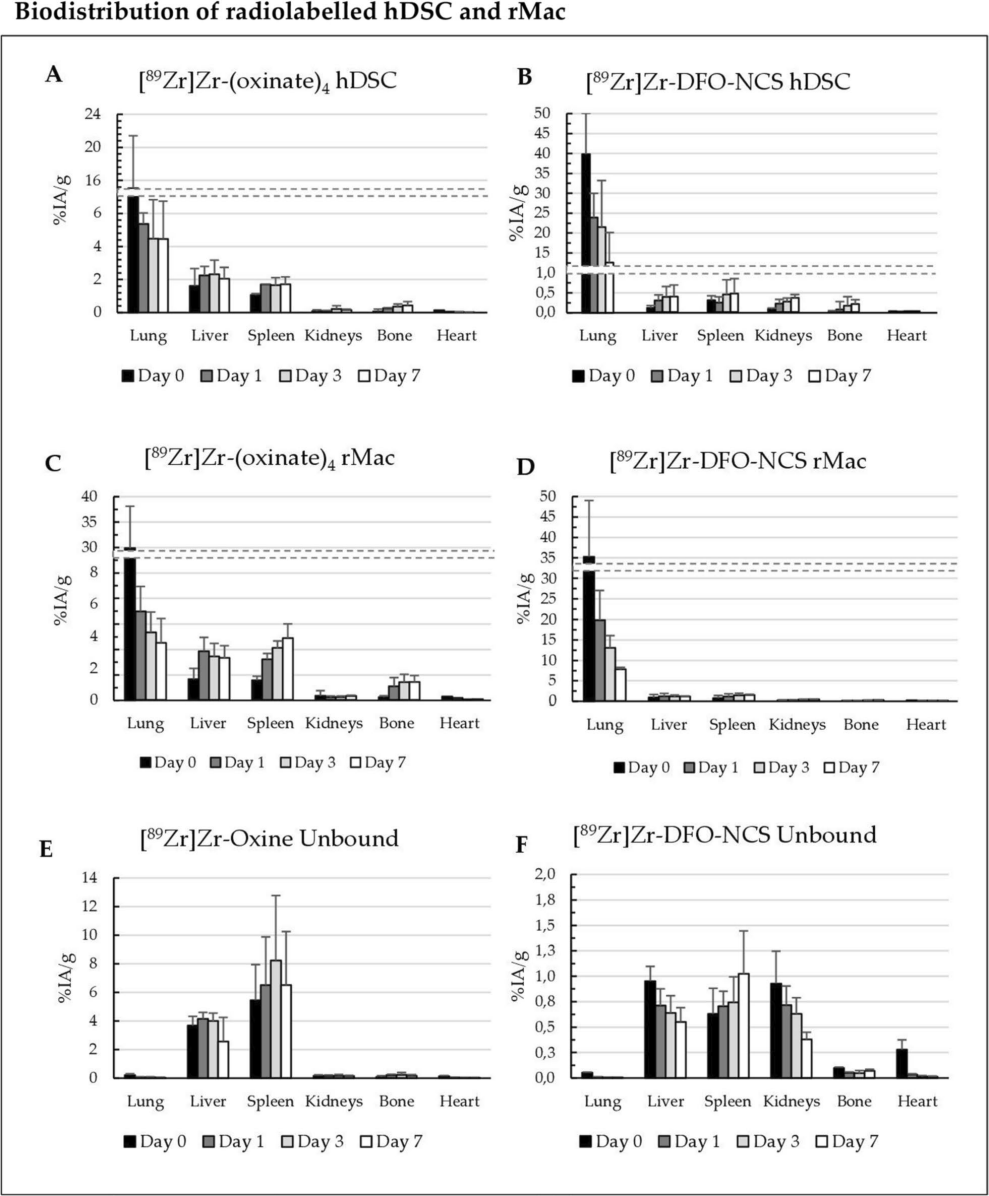
Radioactive biodistribution was decay corrected against the time of injection on day 0 and presented as mean % of the injected activity per gram tissue and standard deviation (%IA/g ± SD). Organ data are collected as the (ROI) from microPET imaging at time points day 0, 1, 3 and 7. Rats received i.v. injections of hDSC or rMac labelled with both radiotracers: **a** [^89^Zr]Zr-(oxinate)_4_ labelled hDSC, **b** [^89^Zr]Zr-DFO-NCS labelled hDSC, **c** [^89^Zr]Zr-(oxinate)_4_ labelled rMac, **d** [^89^Zr]Zr-DFO-NCS labelled rMac, **e** neutralized unbound [^89^Zr]Zr-(oxinate)_4_, **f** neutralized unbound [^89^Zr]Zr-DFO-NCS

Within the first hour after i.v. administration, PET images for both radiotracers and cell types showed an immediate accumulation in the lungs. This behaviour is typical for i.v. injected cells (Figs. 2, 3, 4). What differs between the radiotracers, regardless of what cell type was used, was the fraction that remains in the lungs during the first day as well as the uptake in the liver and spleen over time. Cells labelled with [^89^Zr]Zr-(oxinate)_4_ showed a rapid clearance from the lungs already on the first day, while the [^89^Zr]Zr-DFO-NCS labelled cells seemed to linger in the lungs. The statistically significant difference between the radiotracers was calculated both over time and at each time point by T test and rm ANOVA (Additional files 2, 3, 4: Tables 1, 2 and 3).

Immediately after injection, the lung uptake of [^89^Zr]Zr-(oxinate)_4_ was 15 ± 6.4%IA/g for hDSC and 30 ± 4.3%IA/g for rMac. Somewhat higher accumulation was observed for [^89^Zr]Zr-DFO-NCS with 40 ± 10%IA/g for hDSC (*p* = 0.011) and 35 ± 14%IA/g for rMac (*p* = 0.33) (Additional files 5, 6: Tables 4 and 5). The retention in the lungs for [^89^Zr]Zr-(oxinate)_4_ labelled cells decreased rapidly in the first 24 h, to 5.3 ± 2.7%IA/g for hDSC and 5.5 ± 1.6%IA/g for rMac, at day 1, whereas the [^89^Zr]Zr-DFO-NCS uptake in the lungs remained higher for both cell types, 24 ± 0.61%IA/g for hDSC (*p* = 0.003) and 20 ± 9.0%IA/g for rMac (*p* = 0.045) at day 1. From three days onwards, the uptake of [^89^Zr]Zr-(oxinate)_4_ labelled hDSC continued to clear from in the lungs, 4.5 ± 2.3%IA/g and 4.4 ± 2.3%IA/g on days 3 and 7, respectively. A significantly lower clearance of the lungs from [^89^Zr]Zr-DFO-NCS labelled hDSC continued until day 7 (22 ± 12%IA/g at day 3 and 13 ± 7.6%IA/g at day 7) (*p* = 0.030 and 0.074). A similar difference in clearance of the lungs could be observed from rMac labelled with [^89^Zr]Zr-(oxinate)_4_ (4.2 ± 1.2 and 3.6 ± 1.5%IA/g on days 3 and 7, respectively) compared to [^89^Zr]Zr-DFO-NCS (13 ± 5.7 and 7.7 ± 2.4%IA/g on days 3 and 7, respectively). As the signal cleared from the lung over time, it migrated towards the liver and spleen for both radiotracers (Fig. 4 and Additional file 5: Table 4). [^89^Zr]Zr-(oxinate)_4_ labelled hDSC activity situated in the liver and spleen, with a liver uptake of 2.2 ± 0.96 and 2.0 ± 0.68%IA/g on days 1 and 7 and 1.7 ± 0.53 and 1.7 ± 0.44%IA/g in the spleen on days 1 and 7.

The difference between the tracers in the lung, liver and spleen was less apparent for the rMac labelled cells. [^89^Zr]Zr-(oxinate)_4_ labelled rMac showed a liver uptake of 2.7 ± 0.83 and 2.6 ± 0.77%IA/g on days 3 and 7, respectively. The liver signal for [^89^Zr]Zr-DFO-NCS labelled rMac was 1.1 ± 0.88 and 1.1 ± 0.61%IA/g on days 3 and 7 (*p* = 0.046 and 0.044), respectively. In the spleen, we detected a significant difference between the tracers for rMac on days 1, 3 and 7. The spleen signal from [^89^Zr]Zr-(oxinate)_4_ labelled rMac was 2.6 ± 0.25%IA/g and 3.9 ± 0.90%IA/g on days 1 and 7 and for [^89^Zr]Zr-DFO-NCS labelled rMac was 0.20 ± 0.12%IA/g and 0.11 ± 0.08%IA/g at day 1 and 7 (*p* = 0.042 and 0.024). The accumulation in the kidneys, bone and heart was consistently low for both radiotracers and cell types, except for [^89^Zr]Zr-(oxinate)_4_ labelled rMac.

Control rats injected with unconjugated free neutralized [^89^Zr]Zr-(oxalate)_4_ showed a rapid uptake in the bone within minutes after injection, confirmed by previously published data (Figs. 2, 3) [11, 12]. The uptake in this compartment continued to increase until day 3, after which it appeared to reach saturation and remained unchanged on day 7. The control rats injected with [^89^Zr]Zr-(oxinate)_4_, without cells, showed a rapid migration through the lungs towards the liver and spleen. The uptake in the liver appeared stable from day 0 to day 7 (3.7 ± 0.65 and 2.6 ± 1.7%IA/g on days 0 and 7), while a gradual increase was detected in the spleen (5.5 ± 2.5 and 6.5 ± 3.7%IA/g on days 0 and 7) (Additional file 6: Table 5). Uptake in the lungs, kidneys, bone and heart remained low, less than 1% IA/g, from day 0 up to day 7.

Controls that received hydrolysed [^89^Zr]Zr-DFO-NCS without cells showed an immediate uptake in the liver and kidney following a steady decrease over time (0.95 ± 0.14 and 0.55 ± 0.14%IA/g for the liver, 0.93 ± 0.32 and 0.38 ± 0.07%/IA/g for kidneys on days 0 and 7, respectively). An opposite trend was discovered in spleen uptake which increased over time, measuring 0.63 ± 0.25, 0.74 ± 0.25 and 1.0 ± 0.42%IA/g on days 0, 3 and 7. The majority of the [^89^Zr]Zr-DFO-NCS was quickly excreted through the urine. One-hour post-injection, 33%IA/g was measured in the bladder. Only a small amount of radioactivity could be detected in the lung, bone and heart compartments after the first day, < 0.1%IA/g (Additional file 6: Table 5). There was a significant difference between [^89^Zr]Zr-DFO-NCS radiolabelled rMac and controls in the lungs, liver and spleen, whereas a significant difference was only detected in the lungs and spleen for the radiolabelled hDSC.

The decay-corrected whole-body radioactive retention in rats injected with [^89^Zr]Zr-(oxinate)_4_ labelled cells obtained on day 1 was 90 ± 4.3% for hDSC and 91 ± 6.0% for rMac. The day-1 retention was somewhat lower for [^89^Zr]Zr-DFO-NCS labelled hDSC with 80 ± 15% and for rMac 83 ± 12% (Additional file 5: Table 4). At day 7 a significantly lower amount of radioactivity was left in the body for [^89^Zr]Zr-DFO-NCS labelled hDSC. The radioactive body retention for [^89^Zr]Zr-(oxinate)_4_ labelled cells on day 7 was 74 ± 7.9% for hDSC and 80 ± 7.9% for rMac, while the retention of [^89^Zr]Zr-DFO-NCS labelled cells was only 50 ± 5.0% (*p* = 0.012) and 57 ± 4.9% (*p* = 0.054) for hDSC and rMac, respectively. Hence, both cell lines labelled with [^89^Zr]Zr-DFO-NCS showed a lower total radioactive retention in the animal compared to [^89^Zr]Zr-(oxinate)_4_ labelled cells. The clearance of unbound [^89^Zr]Zr-(oxinate)_4_ was slow, with still 61 ± 12%/IA bound on day 7. Unbound [^89^Zr]Zr-DFO-NCS showed considerably faster clearance with only 10 ± 0.3%/IA left on day 7.

### Dosimetry results

The results indicate, although non-significant, an overall lower effective dose for [^89^Zr]Zr-(oxinate)_4_ labelled cells compared to [^89^Zr]Zr-DFO-NCS (0.24 [0.13–0.35] mSv/MBq and 0.35 [0.23–0.52] mSv/MBq for hDSC, respectively (Table 1). A similar trend could be observed for the rMac labelled cells with an effective dose for [^89^Zr]Zr-(oxinate)_4_ labelled cells compared to [^89^Zr]Zr-DFO-NCS (0.17 [0.13–0.20]mSv/MBq and 0.33 [0.24–0.46]mSv/MBq, respectively). The absorbed doses to lungs were lower for [^89^Zr]Zr-(oxinate)_4_ labelled cells (1.28 [0.43–2.62] mGy/MBq in hDSC and 0.94 [0.71–1.23] in rMac) compared to [^89^Zr]Zr-DFO-NCS labelled cells (2. 72 [1.40–4.39] mGy/MBq in hDSC and 1.86 [0.80–3.0] for rMac). A significant difference was observed in the liver dose from [^89^Zr]Zr-(oxinate)_4_ labelled hDSC (1.18 [1.11–1.35] mGy/MBq) compared to [^89^Zr]Zr-DFO-NCS labelled cells (0.43 [0.39–0.50] mGy/MBq) (*p* =  < 0.001). The same trend was observed for rMac labelled cells, yet not significant, 0.86 [0.51–1.39] mGy/MBq for [^89^Zr]Zr-(oxinate)_4_ labelled rMac and 0.60 [0.49–0.78] mGy/MBq for [^89^Zr]Zr-DFO-NCS labelled rMac (Table 1). The spleen dosage was substantially higher for [^89^Zr]Zr-(oxinate)_4_ labelled compared to [^89^Zr]Zr-DFO-NCS for both cell types. However, due to the small size of the spleen, the dose has little effect on the overall effective dose nor does not cause any significant crossfire. The absorbed dose in kidneys and bone marrow shows comparable results between the radiotracers.

**Table 1:** Absorbed doses (mGy/MBq) to organs and effective dose (mSv/MBq) per administered activity for i.v. administered hDSC and rMac labelled with [^89^Zr]Zr-(oxinate)_4_ or [^89^Zr]Zr-DFO-NCS in rats

Dosimetry: absorbed dose and effective dose from radiolabelled cells				
Organ	hDSC		rMac	
	[^89^Zr]Zr-(oxinate)_4_	[^89^Zr]Zr-DFO-NCS	[^89^Zr]Zr-(oxinate)_4_	[^89^Zr]Zr-DFO-NCS
Lungs *p* value	1.28 [0.43–2.62]	2.72 [1.40–4.39]	0.94 [0.71–1.23]	1.86 [0.80–3.0]
	0.18		0.23	
Liver *p* value	1.18 [1.11–1.35]	0.43 [0.39–0.50]	0.86 [0.51–1.39]	0.60 [0.49–0.78]
	** < 0.001		0.41	
Spleen *p* value	1.38 [1.15–1.64]	0.53 [0.44–0.65]	1.37 [1.11–1.83]	0.83 [0.66–1.16]
	**0.002		0.13	
Kidneys *p* value	0.50 [0033.-0.71]	0.32 [0.22–0.39]	0.42 [0.30–0.56]	0.41 [0.39–0.44]
	0.15		0.78	
Bone marrow *p* value	0.25 [0.12–0.37]	0.27 [0.17–0.40]	0.22 [0.22–0.23]	0.21 [0.14–0.30]
	0.81		0.82	
*Effective dose* *p* value	*0.24 [0.13–0.35]*	*0.35 [0.23–0.52]*	*0.17 [0.13–0.20]*	*0.33 [0.24–0.46]*
	*0.27*		*0.08*	

Values correspond to the arithmetic mean [range] of projected human doses. A statistically significant difference between [^89^Zr]Zr-(oxinate)_4_ and [^89^Zr]Zr-DFO-NCS for the same cell type is marked as **for *p* ≤ 0.01. A *p*-value of ≤ 0.05 was considered statistically significant

## Discussion

Even though cell labelling with PET radiotracers is a promising technique for long-term cell tracking, there is always a risk of affecting the cells. When labelling cells, it is seldom all cells that become radiolabelled, so the difficulty is to assess whether the radiolabelled cells behave the same as the unlabelled cells. The risk of an extracellular labelling method such as [^89^Zr]Zr-DFO-NCS is that it could affect the cells´ interaction with surrounding tissues, hence altering the behaviour and in vivo distribution of the labelled cells. There are few such risks with an intracellular labelling method like [^89^Zr]Zr-(oxinate)_4_; on the other hand, it cannot be excluded that the use of this method could interfere with intercellular mechanisms and alter the cells’ functionality and expression. These risks have to be considered and evaluated individually for each cell type and radiotracer. The effects caused by radiolabelling on hDSC, rMac and PBMC with [^89^Zr]Zr-(oxinate)_4_ and [^89^Zr]Zr-DFO-NCS were previously evaluated in vitro [17]. There it is stated that there was no significant decrease in viability, proliferation and radioactive retention for any of the [^89^Zr] Zr-(oxinate)_4_ labelled cell lines 7 days post-labelling. The same results were seen for [^89^Zr]Zr-DFO-NCS labelled hDSC. Immune cells labelled with [^89^Zr]Zr-DFO-NCS did not show any significant decrease in proliferation or viability. The radioactive retention for [^89^Zr]Zr-DFO-NCS labelled immune cells was significantly lower than cells labelled with [^89^Zr] Zr-(oxinate)_4_, with − 55% and − 25% for rMac and PBMC at day 7, respectively [17]. However, hDSC showed signs of cellular stress, while rMac showed a slight decrease in phagocytosis function.

In this study, we evaluated the short-term (up to 4 days) effects radiolabelling might have on rMac proliferation. For the [^89^Zr]Zr-(oxinate)_4_ labelled cells, radioactive retentions appeared stable from 24 h and no significant difference in cell count compared to controls after 4 days. Cells radiolabelled with [^89^Zr]Zr-DFO-NCS showed a significant decrease in cellular proliferation compared to controls (*p* = 0.03) with no increase in cell count at day 4. The radioactive retention for both [^89^Zr]Zr-(oxinate)_4_ and [^89^Zr]Zr-DFO-NCS labelled cells showed an initial drop during the first 24 h. After which the radioactive loss appeared to stabilize for [^89^Zr]Zr-(oxinate)_4_, while [^89^Zr]Zr-DFO-NCS slowly continued to decrease until day 4. The loss in retention from [^89^Zr]Zr-DFO-NCS is similar to what was observed in our previous study; the drop in retention during the first 24 h is likely due to the loss of cells caused by the radiolabelling procedure. The drop in retention for [^89^Zr]Zr-(oxinate)_4_ is likely due to leakage since there was no significant cell loss. Even though in the previous study the cell dose exceeded the recommended limit for risk of DNA damage, limited damage was detected after 7 days [17]. Here we corrected the radioactive dose between the radiotracers, due to the larger number of cells the dose (MBq/10^6^) to rMac was 1–2 times lower compared to hDSC. Henceforth, the only difference between the same cell line is the use of radiotracer; therefore, we can assume that neither the radioactive dose nor the labelling procedure is the cause of the accumulation in the lungs.

In this study, when comparing [^89^Zr]Zr-(oxinate)_4_ and [^89^Zr]Zr-DFO-NCS, with both rMac and hDSC, we observed different migration patterns of the cells depending on the radiotracer used. Initially, both [^89^Zr]Zr-(oxinate)_4_ and [^89^Zr]Zr-DFO-NCS show a rapid accumulation in the lungs. A large part of the signal from [^89^Zr]Zr-DFO-NCS labelled cells stay in the lung until day 7, while [^89^Zr]Zr-(oxinate)_4_ labelled cells rapidly follow the expected pattern and continue migration to the liver [10, 14, 15, 19, 24].

The control rats injected with unbound [^89^Zr]Zr-(oxinate)_4_ demonstrate a similar biodistribution pattern as the [^89^Zr]Zr-(oxinate)_4_ labelled cells, with high uptake in the spleen and liver. This can complicate the confirmation of the cells’ location without invasive biopsies. We see an almost identical signal in the heart compartment for radiolabelled hDSC compared to the controls with unlabelled [^89^Zr]Zr-(oxinate)_4_. As for the rMac, there is a somewhat prolonged signal in the heart which can be due to the sensitivity of rMac resulting in a slightly larger degree of cell death. It is therefore highly important that the cells are in good condition upon injection since damaged cells will be degraded and the radiotracers might redistribute and confound biodistribution analyses; all injected cells in this study showed 82 ± 6.4% viability upon injection. In the radioactive distribution in control rats that received [^89^Zr]Zr-(oxinate)_4_ labelled cells, we do not see a significant uptake in bone. This indicates that the efflux from dead or damaged cells is not in the form of unbound ^89^Zr. It is more likely that the ^89^Zr is still bound to oxine or conjugated to unspecific structures inside the cell, which is then transported to the liver and spleen instead of bone.

When comparing the controls injected with unbound hydrolysed [^89^Zr]Zr-DFO-NCS with the [^89^Zr]Zr-DFO-NCS labelled cells, they have a different biodistribution. This indicates that it is the cells that have accumulated in the lungs and not just [^89^Zr]Zr-DFO-NCS released from the cell surface. Along with the results from the in vitro stability where 47 ± 14% of the radioactivity is still attached to the cells after 4 days, it is most likely that the majority of signals represent the cells' whereabouts in vivo. We do see a slightly faster lung clearance from rMac labelled with [^89^Zr]Zr-DFO-NCS compared to hDSC, and this could also be explained by a higher degree of cell death due to the sensitivity of rMac. Moreover, dead cells and cell fragments are known to be excreted through the spleen and liver. Since the signals in these organs are hardly detectable, it indicated that the majority of [^89^Zr]Zr-DFO-NCS labelled cells are alive in the lungs. The heart signal from [^89^Zr]Zr-DFO-NCS labelled cells shows a similar pattern as the [^89^Zr]Zr-(oxinate)_4_ labelled cells. These data are based on the ROI of the whole heart, defined without the presence of CT which can result in measurement errors, especially with the proximity to the lungs which can entail a false positive measurement in the heart. Any free ^89^Zr in the blood will rapidly accumulate in the bone; thus, the lack of bone uptake there is hardly any leakage of free ^89^Zr. With the ^89^Zr still bound to unspecific molecules and proteins, the majority of radioactive leakage from dying cells will end up in the liver, spleen and kidneys. The degree of radioactive leakage from [^89^Zr]Zr-DFO-NCS labelled cells would explain why the whole-body retention is lower for rats receiving [^89^Zr]Zr-DFO-NCS labelled cells compared to [^89^Zr]Zr-(oxinate)_4_. Both the loss in whole-body retention and the cellular efflux from radiolabelled rMac after 24 h is 50% higher for [^89^Zr]Zr-DFO-NCS compared to [^89^Zr]Zr-(oxinate)_4_.

When comparing the radioactive dosimetry from radiolabelled cells, the high accumulation of [^89^Zr]Zr-(oxinate)_4_ in both the liver and spleen is compensated by the [^89^Zr]Zr-DFO-NCS high uptake in the lungs. What increases the effective dose for [^89^Zr]Zr-DFO-NCS is the high lung signal which also irradiates surrounding tissues. The proximity of the lungs to the spine, ribcage and shoulders, causes a substantial radioactive crossfire from the lungs to the bone marrow. Although [^89^Zr]Zr-(oxinate)_4_ shows an overall higher %/IA/g uptake in the bone, the high signal from the lungs from [^89^Zr]Zr-DFO-NCS labelled cells increases irradiation of the bone marrow, henceforth the effective dose.

Although conflicting data have been reported, Basal et al. present similar data with high and prolonged uptake in the lungs, which corresponds to our findings [6]. Basal et.al. use a DFO-NCS concentration almost four times higher than in our study. Compared with our study, the only substantial difference between these reports is the concentration of DFO-NCS, while in another study by Lee et. al, they used roughly 800 times lower [^89^Zr]Zr-DFO-NCS concentration and they reported a rapid migration to the liver [15]. It is plausible that a high concentration of [^89^Zr]Zr-DFO-NCS substantially blocks and disrupts essential surface receptors on the cells. If these surface structures are needed for tissue interaction, this might therefore hinder the labelled cells to migrate from the lungs to the liver.

Attempts to lower the concentration and label cells have been proven problematic due to the high loss of [^89^Zr]Zr-DFO-NCS and substantial handling of radioactivity. Since it is difficult to synthesise a stock solution of [^89^Zr]Zr-DFO-NCS with the required concentration and with high specific activity, a larger batch of [^89^Zr]Zr-DFO-NCS is required. If we only take 2% of the [^89^Zr]Zr-DFO-NCS stock solution, we match Lee et al. concentrations of 8.0 pmol DFO-NCS per 5 × 10^6^ cells. The alternative was to increase the number of cells which was not feasible due to the limited harvest per donor. Still, the conflicting reports on the [^89^Zr]Zr-DFO-NCS labelled cells’ behaviour in vivo prove the need for further studies. If this is to be a reliable labelling technique for long-term cell tracking in vivo*,* we have to ensure that the radiolabelled cells mimic the behaviour of the unlabelled cells set for therapy.

## Conclusions

Our results suggest that cell labelling with [^89^Zr]Zr-DFO-NCS may not be reliable or, at the least, consistent with existing data. There are several potential reasons for this multiplicity that causes cells to linger in the lungs, such as (1) extracellular labelling that interferes with cell migration, (2) the accumulation of radioactive cell fragments from dead cells, (3) although no visible evidence was observed in the microscope, cells lumping together due to stress or multiple radiotracer-cell-to-cell binding complexes, or (4) phagocytosis by lymphocytes in the lungs. Whatever reason, this prolonged accumulation in the lungs contributes to the high radiation dose per administered activity for [^89^Zr]Zr-DFO-NCS compared to [^89^Zr]Zr-(oxinate)_4_. Studies to investigate the reasons behind the conflicting behaviour of [^89^Zr]Zr-DFO-NCS labelled cells are still ongoing. For now, our conclusion from these data is that [^89^Zr]Zr-(oxinate)_4_ is more reliable and suitable for long-term cell tracking in vivo due to consistent radiosynthesis and stable cell labelling. The concerning limitation of [^89^Zr]Zr-(oxinate)_4_ is the drop in ^89^Zr retention during the first 24 h; however, studies on protocol optimizations are ongoing to minimize the tracer leakage. The slow clearance of any unbound [^89^Zr]Zr-(oxinate)_4_ can complicate analysis. This novel head-to-head in vivo comparison of the two tracers with the same cell types has shed light on some limitations that have not yet been discussed as well as comparing the biodistribution of the injected radiolabelled cells with control rats receiving only unbound radiotracer, without cells. We are currently conducting studies on proving the exact location of the injected cells; hopefully, this will disclose the reason behind the dislocation of [^89^Zr]Zr-DFO-NCS labelled cells.

### Supplementary Information

**Additional file 1.** Materials and Methods for Production of Zirconium-89; Radiosynthesis of [^89^Zr]Zr-(oxalate)_4_ and [^89^Zr]Zr-DFO-NCS; Radiolabelling of hDSC and rMac; and Dosimetry calculations.

**Additional file 2.** Statistical analysis of cell biodistribution, comparing the two radiotracers for each cell type. Statistical significance was evaluated with rm-ANOVA or t-test. A p-value of ≤ 0.5 was considered statistically significant and marked with* ≤ 0.05 or ** ≤ 0.01.

**Additional file 3.** Statistical analysis of the biodistribution over time between [^89^Zr]Zr-(oxinate)_4_ labelled cells and unbound [^89^Zr]Zr-(oxinate)_4_. Statistical significance was evaluated with rm-ANOVA or t-test. A p-value of ≤ 0.5 was considered statistically significant and marked with* ≤ 0.05 or ** ≤ 0.01.

**Additional file 4.** Statistical analysis of the biodistribution over time between [^89^Zr]Zr-DFO-NCS labelled cells and unbound [^89^Zr]Zr-DFO-NCS. Statistical significance was evaluated with rm-ANOVA or t-test. A p-value of ≤ 0.5 was considered statistically significant and marked with* ≤ 0.05 or ** ≤ 0.01.

**Additional file 5.** Biodistribution of decay-corrected radioactivity presented as % of the injected activity per gram tissue and standard deviation (%IA/g ±SD) in the region of interest (ROI) from micro-PET imaging. Rats received i.v injections with (1) [^89^Zr]Zr-(oxinate)_4_ or [^89^Zr]Zr-DFO-NCS labelled hDSC (2) or rMac. The total whole-body activity is presented as decay-corrected % of the injected activity. Statistical analysis of the biodistribution over time between the different radiotracers was performed for each organ and was evaluated with rm-ANOVA. A p-value of ≤ 0.5 was considered statistically significant and marked with* ≤ 0.05 or ** ≤ 0.01. P-values are shown in (Supplementary Table 1).

**Additional file 6.** Biodistribution of decay-corrected radioactivity presented as % of the injected activity per gram tissue and standard deviation (%IA/g ±SD) in the region of interest (ROI) from micro-PET imaging. Rats received i.v injections with [^89^Zr]Zr-(oxinate)_4_ or [^89^Zr]Zr-DFO-NCS without cells. Statistical analysis of the biodistribution between radiolabeled cells and controls was performed for each organ and evaluated with rm-ANOVA or t-test. A p-value of ≤ 0.5 was considered statistically significant and marked with* ≤ 0.05 or ** ≤ 0.01. P-values are shown in (Supplementary Tables 2 and 3).

## Data Availability

The data presented in this study are available on request to the corresponding authors.

## Abbreviations

CLE: Cell Labelling Efficiency
CT: Computer Chromatography
3D: Three Dimensional
DFO: Deferoxamine
DFO-NCS: P-SCN-Bn-Deferoxamine
DMSO: Dimethyl Sulphoxide
DTPA: Diethylenetriaminepentaacetic Acid
FBS: Foetal Bovine Serum
hDSC: Decidual Stroma Cells
HPLC: High-Pressure Liquid Chromatography
%IA/g: Percentage of Injected Activity per gram tissue
iTLC: Instant Thin-Layer Chromatography
i.v: Intravenously
k/MBq: Kilo/Mega Becquerel
mGy: Milli-Gray
mSv: Milli-Sievert
Na_2_CO_3_: Sodium Carbonate
NaOH: Sodium Acetate Buffer
Oxine or oxinate: 8-Hydroxyquinoline
PET: Positron Emission Tomography
RCY: Radio Chemical Yield
rMac: Rat Macrophages
ROI: Region of Interest
RT: Room Temperature
SD: Standard Deviation
SPECT: Single-Photon Emission Computer Tomography
^89^Zr: Zirconium 89
