# Supplementary material for: Comparative in vivo biodistribution of cells labelled with [89Zr]Zr-(oxinate)4 or [89Zr]Zr-DFO-NCS using PET
Source: EJNMMI Res. 2023 Aug 8;13:73. doi: 10.1186/s13550-023-01021-1 (PMC10409919; doi:10.1186/s13550-023-01021-1)
Supplement: Supplementary file 1 — Additional file 1. Materials and Methods for Production of Zirconium-89; Radiosynthesis of [89Zr]Zr-(oxalate)4 and [89Zr]Zr-DFO-NCS; Radiolabelling of hDSC and rMac; and Dosimetry calculations. [file 13550_2023_1021_MOESM1_ESM.docx]

Comparative *in vivo* biodistribution of cells labelled with [^89^Zr]Zr-(oxinate)_4_ or [^89^Zr]Zr-DFO-NCS using PET

Supplementary

Ida Friberger^1^*, Joachim N. Nilsson^2,3^, Li Lu^1^, Jonathan Siikanen^2,4^, Oscar Ardenfors^2^, Stefan Milton^1,4^, Erik Samén^4,5^, Jeroen A.C.M. Goos^1,4,5^, Mattias Carlsten^6,7^, Staffan Holmin^1,8^ and Thuy A. Tran^1,4,5^

1. Department of Clinical Neuroscience, Karolinska Institutet, Stockholm, Sweden; ida.friberger@ki.se (IF); Li.lu@ki.se (LL); staffan.holmin@ki.se (SH), jeroen.goos@ki.se (JG), stefan.milton@ki.se (SM)
2. Department of Medical Radiation Physics and Nuclear Medicine, Karolinska University Hospital, Stockholm, Sweden; joachim.nilsson@ki.se (JN), jonathan.siikanen@ki.se (JS), (OA) oscar.ardenfors@ki.se
3. Department of Molecular Medicine and Surgery, Karolinska Institutet, Stockholm, Sweden; joachim.nilsson@ki.se (JN)
4. Department of Oncology and Pathology, Karolinska Institutet, Stockholm, Sweden; jonathan.siikanen@ki.se (JS); erik.samen@ki.se (ES); thuy.tran@ki.se (TT), jeroen.goos@ki.se (JG),stefan.milton@ki.se (SM)
5. Department of Radiopharmacy, Karolinska University Hospital, Stockholm, Sweden; erik.samen@ki.se (ES); thuy.tran@ki.se (TT), jeroen.goos@ki.se (JG)
6. Center for Hematology and Regenerative Medicine (HERM), Karolinska Institutet, Stockholm, Sweden; mattias.carlsten@ki.se (MC)
7. Centre for Cell Therapy and Allogeneic Stem Cell Transplantation (CAST), Karolinska Comprehensive Cancer Center, Karolinska University Hospital, Stockholm, Sweden;
8. Department of Neuroradiology, Karolinska University Hospital, Stockholm, Sweden; staffan.holmin@ki.se (SH)

*Corresponding author: Ida Friberger, ida.friberger@ki.se, Tel: +46733-115977, Department of Clinical Neuroscience, Karolinska Institutet, Stockholm, Sweden.

## Materials and Methods

## Production of Zirconium-89

^89^Zr was purchased from PerkinElmer or produced in-house with a cyclotron (PETtrace 800, GE Healthcare) with an ^89^Y(p,n)^89^Zr reaction as previously described [1].

Natural (monoisotopic) yttrium foils of 15.5 mm diameter, 0.25 mm thick were pneumatically transferred to a solid target system (EDS/PTS, Comecer) and irradiated with 25 μA protons in a cyclotron for 1-3 hrs. Proton energy was degraded to a nominal 12.6 MeV to minimize the co-production of long-lived ^88^Zr and ^88^Y. Separation of ^89^Zr from ^89^Y was performed with a separation module (Taddeo PRF, Comecer) using a homemade hydroxamate column as described previously [2,3]. Prior to separation, the column was activated with acetonitrile (TraceSELECT, Honeywell™ Riedel-de-Haën™), H2O (TraceSELECT, Honeywell Riedel-de Haen) and 2 M hydrochloric acid (HCl). The irradiated yttrium foil was dissolved with 6 M HCl (Fisher Scientific) and diluted with H2O. The ^89^Zr solution was loaded onto the resin column and washed with 2 M HCl and H2O. ^89^Zr was then eluted with 1.6 mL, 1 M oxalic acid (Sigma-Aldrich). The experimental saturation yield of [89Zr]Zr-oxalate was 1440 ± 450 MBq/μA (n = 11). The radionuclidic purity (RNP) of ^89^Zr-eluates was determined by gamma spectroscopy using an energy and efficiency calibrated High Purity Germanium detector (Canberra with Cryo-Cycle II Hybrid Cryostat). RNP of ^89^Zr was over 99.99%.

**Radiosynthesis of [^89^Zr]Zr-(oxalate)_4_ and [^89^Zr]Zr-DFO-NCS**

Synthesis of [^89^Zr]Zr-(oxinate)_4_ and [^89^Zr]Zr-DFO-NCS with a radiochemical yield of over 95% was obtained according to our previous publication (in short se supplementary) [1].

In short, aliquots of 5-20 MBq [^89^Zr]Zr-(oxalate)_4_ were mixed with 0.1 M sodium acetate buffer (NaOAc) (Merck Millipore) in a centrifuge tube. Oxine (8-hydroxyquinoline)(Sigma-Aldrich), dissolved in 99% ethanol (Sigma-Aldrich), was then added to the NaOAc mixture and pH was adjusted to 9.1 using 1 M sodium carbonate (Honeywell). The reaction mixture was agitated for 60 minutes at 65°C. The radiochemical yield (RCY) was determined by instant thin-layer chromatography (iTLC) with a 20 mM citric solution mobile phase (AR-2000, Eckert & Ziegler, WinScan software version 3.0). An RCY over 95% was accepted and no further purification was needed for cell labelling.

[^89^Zr]Zr-DFO-NCS was synthesized according to our previously published protocol (1). DFO-NCS (p-SCN-Bn-Deferoxamine) (Macrocyclics) was dissolved in DMSO (Sigma-Aldrich). [^89^Zr]Zr-(oxalate)_4_ was suspended in 0.5 M PBS buffer (Sigma-Aldrich) and neutralized with 1 M sodium carbonate to pH 7.4. Dissolved DFO-NCS was added to the [^89^Zr]Zr-(oxalate)_4_ mixture and incubated for 60 minutes at room temperature. RCY was determined with iTLC with a 50 mM DTPA mobile phase. An RCY of over 95% was accepted with no need for further purification.

**Radiolabelling of hDSC and rMac**

The hDSC and rMac were labelled with [^89^Zr]Zr-(oxinate)_4_ or [^89^Zr]Zr-DFO-NCS according to our previous publication [1]. In short, [^89^Zr]Zr-oxine was neutralized using 1 M oxalic acid and mixed with Tris buffer (Mallinckrodt). Approximately 1-3 MBq/10^6^ cells [^89^Zr]Zr-(oxinate)_4_ were added to cells suspended in PBS, followed by incubation for 40 minutes at 37°C and 5% CO_2_ in an incubator. [^89^Zr]Zr-DFO-NCS was added directly to the PBS cell suspension with a concentration of 1-4 MBq/10^6^ cells and incubated for 40 minutes at room temperature. After incubation, all cells were centrifuged at 183 RCF or 10 minutes and washed with PBS. The supernatants and cell pellets were collected and the amount of remaining radioactivity was measured using a dose calibrator to determine the cell labelling efficiency (CLE). Cell counts and viability were analyzed with a 1:1 cell sample and Trypan Blue staining and quantified using a cell counter (Countess II Automated Cell Counters, Invitrogen). Our previous publication discusses the evaluation of ^89^Zr retention, phagocytic ability and phenotype [1].

**Dosimetry calculations**

For each organ, the time-integrated activity coefficients were calculated by numerical integration of the time-activity curve using mono-exponential or trapezoidal integration. The mono-exponential fit was used when the uptake was rapid (maximum activity concentration reached 30-90 min post-injection) and trapezoidal integration was used for all other cases. Human organ-absorbed doses were consequently calculated using the time-integrated activity coefficients (residence time) in rats determined from the PET data. Using those time-integrated activity coefficients together with human dose-conversion factors derived using IDAC-Dose2.1 with reference organ masses [4], corresponding doses in humans were calculated. Effective doses were calculated for all individuals using tissue-weighting factors according to ICRP publication 103 (ICRP, 2007. The 2007 Recommendations of the International Commission on Radiological Protection. ICRP Publication 103. Ann. ICRP 37 (2-4)). Dosimetric calculations were performed using in-house software for dosimetric analysis of image data (MATLAB), described in a previous publication [5].

**References**

1. Friberger, I.; Jussing, E.; Han, J.; Goos, J.A.C.M.; Siikanen, J.; Kaipe, H.; Lambert, M.; Harris, R.A.; Samén, E.; Carlsten, M.; et al. Optimisation of the Synthesis and Cell Labelling Conditions for [89Zr]Zr-Oxine and [89Zr]Zr-DFO-NCS: A Direct In Vitro Comparison in Cell Types with Distinct Therapeutic Applications. *Mol. Imaging Biol.* **2021**, *23*, 952–962, doi:10.1007/s11307-021-01622-z.

2. Holland, J.P.; Sheh, Y.; Lewis, J.S. Standardized Methods for the Production of High Specific-Activity Zirconium-89. *Nucl. Med. Biol.* **2009**, *36*, 729–739, doi:10.1016/J.NUCMEDBIO.2009.05.007.

3. Verel, I.; Visser, G.W.M.; Boellaard, R.; Stigter-van Walsum, M.; Snow, G.B.; van Dongen, G.A.M.. 89Zr Immuno-PET: Comprehensive Procedures for the Production of 89Zr-Labeled Monoclonal Antibodies. *J. Nucl. Med.* **2003**, *44*.

4. Andersson, M.; Johansson, L.; Eckerman, K.; Mattsson, S. IDAC-Dose 2.1, an Internal Dosimetry Program for Diagnostic Nuclear Medicine Based on the ICRP Adult Reference Voxel Phantoms. *EJNMMI Res.* **2017**, *7*, doi:10.1186/S13550-017-0339-3.

5. Ardenfors, O.; Nilsson, J.N.; Thor, D.; Hindorf, C. Simplified Dosimetry for Kidneys and Tumors in 177Lu-Labeled Peptide Receptor Radionuclide Therapy. *EJNMMI Phys.* **2022**, *9*, doi:10.1186/S40658-022-00473-Z.
