# Supplementary material for: Comparative in vivo biodistribution of cells labelled with [89Zr]Zr-(oxinate)4 or [89Zr]Zr-DFO-NCS using PET
Source: EJNMMI Res. 2023 Aug 8;13:73. doi: 10.1186/s13550-023-01021-1 (PMC10409919; doi:10.1186/s13550-023-01021-1)
Supplement: Supplementary file 2 — Additional file 2. Statistical analysis of cell biodistribution, comparing the two radiotracers for each cell type. Statistical significance was evaluated with rm-ANOVA or t-test. A p-value of ≤ 0.5 was considered statistically significant and marked with* ≤ 0.05 or ** ≤ 0.01. [file 13550_2023_1021_MOESM2_ESM.docx]

**Supplementary Table 1, p-values for in vivo biodistribution of radiolabelled cells**

| **P-values from statistical analysis of *in vivo* biodistribution** | | | | | | | |
| --- | --- | --- | --- | --- | --- | --- | --- |
| **[^89^Zr]Zr-(oxinate)_4_ versus [^89^Zr]Zr-DFO-NCS (p-values) hDSC** | | | | | | | |
| **Organ** | **Day 0** | **Day 1** | | **Day 3** | **Day 7** | | **P-value over time** |
| Lungs | 0.011* | 0.003** | 0.030* | | | 0.074 | 0.34 |
| Liver | 0.011* | 0.016* | 0.012* | | | 0.011* | 0.11 |
| Spleen | 0.052 | 0.006** | 0.014* | | | 0.011* | 0.31 |
| Kidneys | 0.30 | 0.055 | 0.37 | | | 0.009** | 0.20 |
| Bone | 0.041* | 0.084 | 0.081 | | | 0.12 | 0.074 |
| Heart | 0.004** | 0.020* | 0.095 | | | 0.43 | 0.22 |
| Total whole-body activity | 1 | 0.15 | 0.060 | | | *0.012 | *0.043 |
| **[^89^Zr]Zr-(oxinate)_4_ versus [^89^Zr]Zr-DFO-NCS rMac** | | | | | | | |
| **Organ** | **Day 0** | **Day 1** | | **Day 3** | **Day 7** | | **P-value over time** |
| Lungs | 0.33 | 0.045* | 0.050* | | | 0.053 | 0.57 |
| Liver | 0.27 | 0.045* | 0.046* | | | 0.044* | 0.39 |
| Spleen | 0.16 | 0.042* | 0.033* | | | 0.024* | 0.56 |
| Kidneys | 0.33 | 0.13 | 0.037* | | | 0.016* | 0.61 |
| Bone | 0.066 | 0.059 | 0.025* | | | 0.017* | 0.076 |
| Heart | 0.22 | 0.011* | 0.066 | | | 0.071 | 0.13 |
| Total whole-body activity | 1 | 0.23 | 0.089 | | | 0.054 | 0.073 |

Statistical analysis of cell biodistribution, comparing the two radiotracers for each cell type. Statistical significance was evaluated with rm-ANOVA or t-test. A p-value of <0.05 was considered statistically significant and marked with * = <0.05 or ** = <0.01.
