# Supplementary material for: Comparative in vivo biodistribution of cells labelled with [89Zr]Zr-(oxinate)4 or [89Zr]Zr-DFO-NCS using PET
Source: EJNMMI Res. 2023 Aug 8;13:73. doi: 10.1186/s13550-023-01021-1 (PMC10409919; doi:10.1186/s13550-023-01021-1)
Supplement: Supplementary file 3 — Additional file 3. Statistical analysis of the biodistribution over time between [89Zr]Zr-(oxinate)4 labelled cells and unbound [89Zr]Zr-(oxinate)4. Statistical significance was evaluated with rm-ANOVA or t-test. A p-value of ≤ 0.5 was considered statistically significant and marked with* ≤ 0.05 or ** ≤ 0.01. [file 13550_2023_1021_MOESM3_ESM.docx]

**Supplementary Table 2,** **p-values for in vivo biodistribution of [^89^Zr]Zr-(oxinate)_4_**

| **P-values from statistical analysis of *in vivo* biodistribution** | | | | | | | |
| --- | --- | --- | --- | --- | --- | --- | --- |
| **Unbound [^89^Zr]Zr-(oxinate)_4_ versus [^89^Zr]Zr-(oxinate)_4_ labelled hDSC** | | | | | | | |
| **Organ** | **Day 0** | **Day 1** | | **Day 3** | **Day 7** | | **P-value over time** |
| Lungs | *0.026 | *0.040 | *0.046 | | | *0.043 | *0.047 |
| Liver | *0.026 | *0.028 | *0.019 | | | *0.014 | 0.16 |
| Spleen | 0.050 | *0.012 | **0.009 | | | **0.007 | 0.81 |
| Kidneys | *0.014 | *0.015 | 0.16 | | | *0.011 | 0.37 |
| Bone | *0.037 | 0.080 | *0.032 | | | 0.052 | 0.17 |
| Heart | **0.007 | **0.004 | **0.006 | | | *0.066 | 0.32 |
| **Unbound [^89^Zr]Zr-(oxinate)_4_ versus [^89^Zr]Zr-(oxinate)_4_ labelled rMac** | | | | | | | |
| **Organ** | **Day 0** | **Day 1** | | **Day 3** | **Day 7** | | **P-value over time** |
| Lungs | *0.036 | *0.038 | *0.041 | | | 0.080 | 0.34 |
| Liver | 0.12 | *0.038 | *0.044 | | | *0.040 | 0.15 |
| Spleen | *0.022 | *0.011 | **0.009 | | | *0.026 | 0.27 |
| Kidneys | 0.37 | 0.11 | 0.070 | | | *0.023 | 0.15 |
| Bone | 0.12 | 0.16 | 0.085 | | | 0.060 | 0.41 |
| Heart | 0.16 | *0.036 | 0.10 | | | 0.120 | 0.10 |

Statistical analysis of the biodistribution over time between [^89^Zr]Zr-(oxinate)_4_ labelled cells and unbound [^89^Zr]Zr-(oxinate)_4_. Statistical significance was evaluated with rm-ANOVA or t-test. A p-value of <0.05 was considered statistically significant and marked with * = <0.05 or ** = <0.01
