# Supplementary material for: Comparative in vivo biodistribution of cells labelled with [89Zr]Zr-(oxinate)4 or [89Zr]Zr-DFO-NCS using PET
Source: EJNMMI Res. 2023 Aug 8;13:73. doi: 10.1186/s13550-023-01021-1 (PMC10409919; doi:10.1186/s13550-023-01021-1)
Supplement: Supplementary file 4 — Additional file 4. Statistical analysis of the biodistribution over time between [89Zr]Zr-DFO-NCS labelled cells and unbound [89Zr]Zr-DFO-NCS. Statistical significance was evaluated with rm-ANOVA or t-test. A p-value of ≤ 0.5 was considered statistically significant and marked with* ≤ 0.05 or ** ≤ 0.01. [file 13550_2023_1021_MOESM4_ESM.docx]

**Supplementary Table 3, p-values for in vivo biodistribution of [^89^Zr]Zr-DFO-NCS**

| **P-values from statistical analysis of *in vivo* biodistribution** | | | | | | | |
| --- | --- | --- | --- | --- | --- | --- | --- |
| **Unbound [^89^Zr]Zr-DFO-NCS versus [^89^Zr]Zr-DFO-NCS labelled hDSC** | | | | | | | |
| **Organ** | **Day 0** | **Day 1** | | **Day 3** | **Day 7** | | **P-value over time** |
| Lungs | *0.032 | *0.031 | 0.12 | | | 0.14 | *0.031 |
| Liver | 0.15 | 0.11 | 0.18 | | | 0.20 | 0.08 |
| Spleen | 0.068 | 0.15 | 0.24 | | | 0.22 | 0.05 |
| Kidneys | 0.094 | 0.10 | 0.060 | | | *0.027 | 0.11 |
| Bone | 0.15 | 0.22 | 0.071 | | | 0.050 | 0.11 |
| Heart | 0.49 | 0.30 | 0.17 | | | *0.018 | 0.23 |
| **Unbound [^89^Zr]Zr-DFO-NCS versus [^89^Zr]Zr-DFO-NCS labelled rMac** | | | | | | | |
| **Organ** | **Day 0** | **Day 1** | | **Day 3** | **Day 7** | | **P-value over time** |
| Lungs | 0.068 | 0.089 | 0.084 | | | *0.044 | 0.070 |
| Liver | 0.26 | 0.21 | 0.16 | | | 0.13 | 0.30 |
| Spleen | 0.28 | 0.21 | 0.19 | | | 0.14 | 0.09 |
| Kidneys | 0.13 | *0.020 | *0.013 | | | **0.004 | 0.067 |
| Bone | 0.16 | *0.048 | *0.036 | | | *0.022 | 0.073 |
| Heart | 0.18 | 0.093 | 0.16 | | | 0.14 | 0.15 |

Statistical analysis of the biodistribution over time between [^89^Zr]Zr-DFO-NCS labelled cells and unbound [^89^Zr]Zr-DFO-NCS. Statistical significance was evaluated with rm-ANOVA or t-test. A p-value of <0.05 was considered statistically significant and marked with * = <0.05 or ** = <0.01.
