# Supplementary material for: Comparative in vivo biodistribution of cells labelled with [89Zr]Zr-(oxinate)4 or [89Zr]Zr-DFO-NCS using PET
Source: EJNMMI Res. 2023 Aug 8;13:73. doi: 10.1186/s13550-023-01021-1 (PMC10409919; doi:10.1186/s13550-023-01021-1)
Supplement: Supplementary file 5 — Additional file 5. Biodistribution of decay-corrected radioactivity presented as % of the injected activity per gram tissue and standard deviation (%IA/g ±SD) in the region of interest (ROI) from micro-PET imaging. Rats received i.v injections with (1) [89Zr]Zr-(oxinate)4 or [89Zr]Zr-DFO-NCS labelled hDSC (2) or rMac. The total whole-body activity is presented as decay-corrected % of the injected activity. Statistical analysis of the biodistribution over time between the different radiotracers was performed for each organ and was evaluated with rm-ANOVA. A p-value of ≤ 0.5 was considered statistically significant and marked with* ≤ 0.05 or ** ≤ 0.01. P-values are shown in (Supplementary Table 1). [file 13550_2023_1021_MOESM5_ESM.docx]

| **Radioactive *in vivo* biodistributions of radiolabeled cells** | | | | | | | | |
| --- | --- | --- | --- | --- | --- | --- | --- | --- |
| **[^89^Zr]Zr-(oxinate)_4_ labelled hDSC** | | | | | **[^89^Zr]Zr-DFO-NCS labelled hDSC** | | | |
|  | **Day 0** | **Day 1** | **Day 3** | **Day 7** | **Day 0** | **Day 1** | **Day 3** | **Day 7** |
| Lung  ±SD | *15  ±6.4% | **5.3  ±2.7% | 4.5  ±2.3% | 4.4  ±2.3% | *40  ±10% | **24  ±0.61% | 22  ±12% | 13  ±7.6% |
| Liver  ±SD | *1.6  ±0.66% | *2.2  ±0.96% | *2.3  ±0.85% | **2.0  ±0.68% | *0.11  ±0.06% | *0.29  ±0.15% | *0.39  ±27% | **0.40  ±30% |
| Spleen  ±SD | 1.1  ±0.54% | **1.7  ±0.53% | *1.6  ±0.46% | **1.7  ±0.44% | 0.30  ±0.11% | **0.24  ±15% | *0.45  ±28% | **0.48  ±38% |
| Kidney  ±SD | 0.09  ±0.03% | 0.09  ±0.03% | 0.21  ±0.20% | *0.15  ±0.05% | 0.07  ±0.03% | 0.22  ±0.11% | 0.26  ±0.10% | *0.36  ±0.09% |
| Bone  ±SD | *0.07  ±0.03% | 0.26  ±0.17% | 0.35  ±0.16% | *0.42  ±0.23% | *0.02  ±0.01% | 0.06  ±0.20% | 0.16  ±0.23% | *0.21  ±0.11% |
| Heart  ±SD | **0.14  ±0.03% | *0.05  ±0.01% | 0.04  ±0.01% | 0.02  ±0.01% | **0.02  ±0.02% | *0.01  ±0.01% | 0.02  ±0.01% | 0.02  ±0.004% |
| Total whole-body activity | 100% | 90  ±4.3% | 83  ±4.7% | *74  ±7.9% | 100 % | 80  ±15% | 69  ±10% | *50  ±5.0% |
| **[^89^Zr]Zr-(oxinate)_4_ labelled rMac** | | | | | **[^89^Zr]Zr-DFO-NCS labelled rMac** | | | |
|  | **Day 0** | **Day 1** | **Day 3** | **Day 7** | **Day 0** | **Day 1** | **Day 3** | **Day 7** |
| Lung  ±SD | 30  ±4.3% | *5.5  ±1.6% | *4.2  ±1.3% | 3.6  ±1.5% | 35  ±14% | *20  ±9.0% | *13  ±5.7% | 7.7  ±2.4% |
| Liver  ±SD | 1.3  ±0.68% | *3.1  ±0.86% | *2.7  ±0.83% | *2.6  ±0.77% | 0.82  ±0.74% | *1.1  ±0.88% | *1.1  ±0.68% | *1.1  ±0.61% |
| Spleen  ±SD | 1.2  ±0.25% | *2.6  ±0.25% | *3.3  ±0.44% | *3.9  ±0.90% | 0.68  ±0.65% | *1.1  ±0.83% | *1.4  ±0.98% | *1.4  ±0.84% |
| Kidney  ±SD | 0.26  ±0.32% | 0.17  ±0.08% | *0.18  ±0.07% | *0.24  ±0.05% | 0.15  ±0.08% | 0.26  ±0.05% | *0.33  ±0.05% | *0.39  ±0.03% |
| Bone  ±SD | 0.17  ±0.09% | 0.86  ±0.54% | *1.1  ±0.49% | *1.1  ±0.41% | 0.04  ±0.03% | 0.10  ±0.03% | *0.15  ±0.04% | *0.21  ±0.04% |
| Heart  ±SD | 0.22  ±0.14% | *0.13  ±0.04% | 0.04  ±0.02% | 0.05  ±0.03% | 0.12  ±0.08% | *0.03  ±0.01% | 0.01  ±0.01% | 0.01  ±0.01% |
| Total whole-body activity | 100% | 91  ±6.0% | 86  ±4.6% | 80  ±7.9% | 100% | 83%  ±12 | 68%  ±9.1 | 57  ±4.9% |

**Supplementary Table 4, *In vivo* biodistributions (%IA/g) of radiolabeled cells**

Biodistribution of decay-corrected radioactivity presented as % of the injected activity per gram tissue and standard deviation (%IA/g ±SD) in the region of interest (ROI) from micro-PET imaging. Rats received i.v injections with **(1)** [^89^Zr]Zr-(oxinate)_4_ or [^89^Zr]Zr-DFO-NCS labelled hDSC **(2)** or rMac. The total whole-body activity is presented as decay-corrected % of the injected activity. Statistical analysis of the biodistribution over time between the different radiotracers was performed for each organ and was evaluated with rm-ANOVA. A p-value of <0.05 was considered statistically significant and marked with * = <0.05 or ** = <0.01. P-values are shown in (Supplementary Table 1).
