# Supplementary material for: Comparative in vivo biodistribution of cells labelled with [89Zr]Zr-(oxinate)4 or [89Zr]Zr-DFO-NCS using PET
Source: EJNMMI Res. 2023 Aug 8;13:73. doi: 10.1186/s13550-023-01021-1 (PMC10409919; doi:10.1186/s13550-023-01021-1)
Supplement: Supplementary file 6 — Additional file 6. Biodistribution of decay-corrected radioactivity presented as % of the injected activity per gram tissue and standard deviation (%IA/g ±SD) in the region of interest (ROI) from micro-PET imaging. Rats received i.v injections with [89Zr]Zr-(oxinate)4 or [89Zr]Zr-DFO-NCS without cells. Statistical analysis of the biodistribution between radiolabeled cells and controls was performed for each organ and evaluated with rm-ANOVA or t-test. A p-value of ≤ 0.5 was considered statistically significant and marked with* ≤ 0.05 or ** ≤ 0.01. P-values are shown in (Supplementary Tables 2 and 3). [file 13550_2023_1021_MOESM6_ESM.docx]

| **Radioactive *in vivo* biodistributions of unbound compounds** | | | | | | | | |
| --- | --- | --- | --- | --- | --- | --- | --- | --- |
| **[^89^Zr]Zr-(oxinate)_4_ controls no cells** | | | | | **[^89^Zr]Zr-DFO-NCS controls no cells** | | | |
|  | **Day 0** | **Day 1** | **Day 3** | **Day 7** | **Day 0** | **Day 1** | **Day 3** | **Day 7** |
| Lung  ±SD | 0.22  ±0.09% | 0.07  ±0.04% | 0.06  ±0.03% | 0.04  ±0.02% | 0.05  ±0.01% | 0.01  ±0.001% | <0.01  ±0.001% | 0.005  ±0.001% |
| Liver  ±SD | 3.7  ±0.65% | 4.2  ±0.45% | 4.0  ±0.56% | 2.6  ±1.7% | 0.95  ±0.14% | 0.71  ±0.16% | 0.64  ±0.17% | 0.55  ±0.14% |
| Spleen  ±SD | 5.5  ±2.5% | 6.5  ±3.4% | 8.2  ±4.6% | 6.5  ±3.7% | 0.63  ±0.25% | 0.71  ±0.15% | 0.74  ±0.25% | 1.02  ±0.42% |
| Kidney  ±SD | 0.16  ±0.07% | 0.17  ±0.05% | 0.17  ±0.09% | 0.15  ±0.05% | 0.93  ±0.32% | 0.72  ±0.19% | 0.63  ±0.16% | 0.38  ±0.07% |
| Bone  ±SD | 0.08  ±0.09% | 0.21  ±0.07% | 0.24  ±0.16% | 0.16  ±0.09% | 0.10  ±0.008% | 0.05  ±0.008% | 0.05  ±0.03% | 0.07  ±0.01% |
| Heart  ±SD | 0.14  ±0.05% | 0.05  ±0.01% | 0.03  ±0.001% | 0.03  ±0.01% | 0.28  ±0.09% | 0.03  ±0.008% | 0.01  ±0.01% | <0.01  ±0.007% |

**Supplementary Table 5, *In vivo* biodistributions (%IA/g) of [^89^Zr]Zr-(oxinate)_4_ and [^89^Zr]Zr-DFO-NCS**

Biodistribution of decay-corrected radioactivity presented as % of the injected activity per gram tissue and standard deviation (%IA/g ±SD) in the region of interest (ROI) from micro-PET imaging. Rats received i.v injections with [^89^Zr]Zr-(oxinate)_4_ or [^89^Zr]Zr-DFO-NCS without cells. Statistical analysis of the biodistribution between radiolabeled cells and controls was performed for each organ and evaluated with rm-ANOVA or t-test. A p-value of <0.05 was considered statistically significant and marked with * = <0.05 or ** = <0.01. P-values are shown in (Supplementary Table 2 and 3).
